# White matter hyperintensity, parent artery steno‐occlusion, and neurological deterioration in anterior circulation single subcortical infarction patients

**DOI:** 10.1002/brb3.3523

**Published:** 2024-05-15

**Authors:** Yunhe Luo, Daosheng Wang, Yiming Jia, Xin Gu, Yuhan Zang, Zhengbao Zhu, Jin Zheng, Ligang Huang, Jing Zhao

**Affiliations:** ^1^ Department of Neurology Minhang Hospital Fudan University Shanghai China; ^2^ Department of Neurosurgery Minhang Hospital Fudan University Shanghai China; ^3^ Department of Epidemiology, School of Public Health and Jiangsu Key Laboratory of Preventive and Translational Medicine for Geriatric Diseases Medical College of Soochow University Suzhou China; ^4^ Department of Neurology Shanghai Sixth People's Hospital, Shanghai Jiao Tong University Shanghai China; ^5^ Shanghai Neurological Rare Disease Biobank and Precision Diagnostic Technical Service Platform Shanghai China

**Keywords:** acute ischemic stroke, neurological deterioration, parent artery steno‐occlusion, single subcortical infarction, white matter hyperintensity

## Abstract

**Background:**

The evidence for the association between white matter hyperintensity (WMH) severity and neurological deterioration (ND) in patients with single subcortical infarction (SSI) remains unclear and whether the association between them is modified by anterior circulation parent artery steno‐occlusion (PAS) is unknown. Herein, we aimed to prospectively investigate the internal relevance.

**Methods:**

In this prospective study, the severity of WMH and PAS were assessed in 288 consecutive patients with anterior circulation SSI arriving at our hospital, a tertiary teaching hospital affiliated with Fudan University, 24 h after onset from January 2017 to December 2018. The multivariable logistic regression model was used to estimate the association between WMH severity and the risk of ND within 7 days after stroke onset as well as the interactive effect between WMH severity and PAS on ND among patients with SSI.

**Results:**

PAS modified the association between WMH severity and ND among patients with SSI (*p*_interaction_ = .029). After multivariate adjustment, the odds ratios of moderate‐severe WMH associated with ND were 1.61 (95% CI, 0.50–5.19; *p*_trend_ = .428) for patients with PAS, and 0.37 (95% CI, 0.14–0.97; *p*_trend_ = .043) for those without PAS. Adding WMH severity to conventional risk factors improved risk prediction for ND in patients without PAS (net reclassification improvement: 48.2%, *p *= .005; integrated discrimination index: 2.5%, *p *= .004) but not in those with PAS.

**Conclusion:**

There was a modified effect of PAS on the association between WMH severity and ND within 7 days after stroke onset among patients with anterior circulation SSI, which deserves more research attention. WMH was negatively associated with ND in anterior circulation SSI patients without PAS.

## Graphical abstract

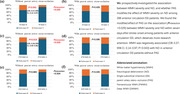

## 1 INTRODUCTION

Single subcortical infarction (SSI), an imaging subtype that partially overlaps with lacunar infarction, accounts for approximately a quarter of ischemic stroke, and over 20% of patients with SSI experience neurological deterioration in the acute period (Del Bene et al., 2012; Kim & Kim, 2014; Kim & Yoon, 2013; Kwapong et al., 2021). SSI is a heterogeneous condition, which may be caused by atherosclerotic artery steno‐occlusion, small vessel disease, and the occlusion of perforators caused by parent artery atherothrombosis with no luminal narrowing (Yoon et al., 2013). Artery stenosis can be easily identified by images, such as computed tomography angiography (CTA), magnetic resonance angiography (MRA), and digital subtraction angiography (Stary et al., 1995). Small vessel disease can be partially reflected by white matter lesions (Cannistraro et al., 2019; Hilal et al., 2017; Wardlaw et al., 2013). For parent artery atherothrombosis with no luminal narrowing, even if the current high‐resolution vessel wall magnetic resonance is used, the contradiction between signal‐to‐noise ratio and resolution contributes to the difficulty in identifying without omission (Smith & Beaudin, 2018), which may lead to false negatives of perforating atherosclerotic lesions.

In the past few years, many studies have been conducted to find some good predictors for ND in patients with SSI, which is highly desirable to aid in decisions regarding stroke care and management. Some researchers have found that distal SSI (an infarction that exists only in the distal area that does not meet the basal surface of the parent artery) was associated with lower rate of ND and more severe periventricular white matter hyperintensity (WMH) compared with proximal SSI (an infarction located adjacent to the parent artery and extending toward the basal surface of the parent artery) (Nam et al., 2021; Yamamoto et al., 2011; Zhang et al., 2014), for distal SSI may be caused by fibrinoid degeneration or lipohyalinosis of small vessels (Caplan, 1989). In consideration of the above‐mentioned findings, WMH, regarded as a neuroimaging marker of small vessel disease (Cannistraro et al., 2019; Hilal et al., 2017), may also have a correlation with ND in patients with SSI. However, some studies investigating the association between WMH and ND in SSI patients have not demonstrated a significant correlation (Lee et al., 2017; Zhang et al., 2014).

After reviewing the literature, we discovered that anterior circulation parent artery steno‐occlusion (PAS), a clear predictor for ND (Etherton et al., 2019; Nam et al., 2021; Park et al., 2015), has been established as a vital role in the modification of the severity of white matter lesions (Lee et al., 2011; Park et al., 2015; Zhai et al., 2018), which might bias the risk prediction of ND using WMH severity in patients with anterior circulation SSI. Hence, we aimed to prospectively investigate the association between WMH severity and ND and to investigate whether PAS modifies the effect of WMH severity on ND among anterior circulation SSI patients.

## 2 MATERIALS AND METHODS

### 2.1 Study design and participants

We prospectively collected patients with anterior circulation SSI within 24 h of onset in our hospital from January 2017 to December 2018. A diagnosis of anterior circulation SSI was based on magnetic resonance imaging, including any size of single subcortical infarction in anterior circulation in the lenticulostriate arterial territory (lentiform nucleus, corona radiata, and internal capsule) (Kim & Yoon, 2013; Nam et al., 2021). A total of 380 potentially eligible patients were enrolled. The additional exclusion criteria of this analysis were as follows: (1) thrombolysis and thrombectomy for treatment (*n* = 29); (2) having atrial fibrillation, atrial flutter, or rheumatic heart disease based on medical history, 24‐h Holter monitoring, and transthoracic echocardiography(*n* = 24); (3) having intracranial vascular dissection (*n* = 1); (4) having vasculitis (*n* = 1); (5) lack of intracranial vascular imaging (*n* = 37). As a result, 288 patients with anterior circulation SSI were finally included in this study.

This study was approved by the Ethics Committee of our hospital (2021‐052‐01K), and written consent was obtained from all study participants or their immediate family members.

### 2.2 Data collection and outcome assessment

Data on demographic characteristics, lifestyle risk factors, and medical history were collected at admission. Information on these factors was obtained by interviews with patients or their family members (if patients were not able to communicate). Routine laboratory determinations (plasma glucose, low‐density lipoprotein cholesterol, creatinine, blood urea nitrogen, hemoglobin, uric acid, etc.) were performed for all enrolled patients using standard procedures at admission. Stroke severity was assessed using the National Institutes of Health Stroke Scale (NIHSS) by trained neurologists at baseline.

All the images were analyzed by two trained neurologists who were blind to clinical features and study outcomes. In case of disagreement, images were reviewed until a consensus was reached.

PAS was assessed at the site of the most severe degree of stenosis by CTA or MRA using established criteria (Owen et al., 2000). The M1 segment of the middle cerebral artery (parent artery of lenticulostriate artery) and the C7 segment of the internal carotid artery (parent artery of anterior choroidal artery) on the side ipsilateral to the lesion were assessed and classified as no luminal narrowing or PAS (any amount of stenosis and occlusion), which was defined as any visible rough or interrupted lumen alteration on MRA or CTA images (Jang et al., 2020).

WMH was evaluated using the Fazekas rating scale with periventricular WMH (PWMH) and deep WMH (DWMH) ratings separately with the magnetic resonance images (mainly T2 FLAIR signal). PWMH was graded as 0 = absence, 1 = “caps” or pencil‐thin lining, 2 = smooth “halo”, and 3 = irregular PWMH extending into the deep white matter. DWMH were rated as 0 = absence, 1 = punctate foci, 2 = beginning confluence of foci, and 3 = large confluent areas (Zhu et al., 2020). We dichotomized PWMH and DWMH to none‐mild (Fazekas score: 0–1) and moderate‐severe (Fazekas score: 2–3). Furthermore, we calculated the total Fazekas score as the sum of PWMH and DWMH, ranging from 0–6. The severity of WMH was categorized as follows: none‐mild WMH (total Fazekas scale score 0 to 2) and moderate‐severe WMH (total Fazekas scale score 3 to 6) (Fazekas et al., 1987; Helenius et al., 2016; Patti et al., 2016).

Study participants were investigated during the first week after anterior circulation SSI onset by experienced neurologists. The study outcome was ND (an increase of ≥2 in the total National Institutes Health Stroke Scale [NIHSS] score or ≥1 in the motor items of NIHSS) within 7 days after anterior circulation SSI (Lee et al., 2017).

### 2.3 Statistical analysis

Baseline characteristics were compared between patients with and without ND in 7 days using analysis of Student's *t*‐test or Wilcoxon rank‐sum test for continuous variables and chi‐squared test for categorical variables. A multivariable logistic regression model was used to estimate the association of WMH severity (including PWMH and DWMH) with ND and to evaluate the effects of WMH severity on ND in patients with and without PAS. Odds ratio (OR) and 95% confidence interval (95% CI) were calculated for the patients with moderate–severe WMH compared with those with none‐mild WMH. Important covariates for ND including age, sex, current smoking, alcohol consumption, baseline NIHSS score at admission, modified Rankin Scale score at onset, history of hypertension, history of diabetes mellitus, history of hyperlipidemia, and history of ischemic stroke were selected based on our prior knowledge and were adjusted in multivariable analyses. In addition, heterogeneity of the ND according to WMH severity was assessed by adding an interaction term (WMH severity×PAS) in the logistic regression models.

Net reclassification improvement (NRI) and integrated discrimination improvement (IDI) were two indexes to assess improvement in model performance accomplished by adding new markers (Pencina et al., 2008). All conventional risk factors (aforementioned potential covariates) were directly incorporated into the model when we calculated NRI and IDI. We constructed a conventional model (only including conventional risk factors) and three new models (adding WMH, PWMH, or DWMH to conventional risk factors) by logistic regression model. To assess the improvement of these three new models in risk prediction for ND after anterior circulation SSI, we calculated NRI and IDI by comparing these models with the conventional model. Two‐tailed *p* < .05 was considered to be statistically significant. All analyses were performed using SAS statistical software (version 9.4, Cary, NC).

## 3 RESULTS

### 3.1 Baseline characteristics

**FIGURE 1:**
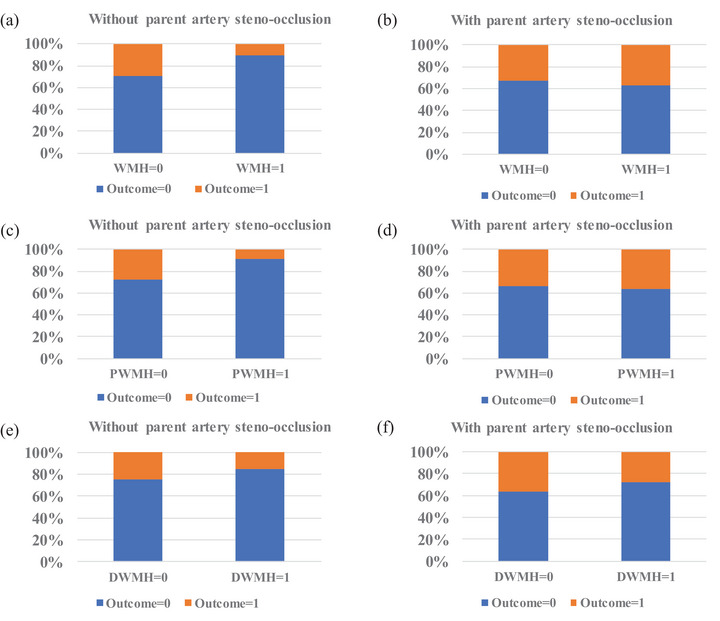
Risk of neurological deterioration according to white matter hyperintensity severity in single subcortical infarction patients with or without parent artery steno‐occlusion. (a) Distribution of outcome according to white matter hyperintensity (WMH) severity in patients without parent artery steno‐occlusion; (b) distribution of outcome according to WMH severity in patients with parent artery steno‐occlusion; (c) distribution of outcome according to periventricular white matter hyperintensity (PWMH) severity in patients without parent artery steno‐occlusion; (d) distribution of outcome according to PWMH severity in patients with parent artery steno‐occlusion; (e) distribution of outcome according to deep white matter hyperintensity (DWMH) severity in patients without parent artery steno‐occlusion; (f) distribution of outcome according to DWMH severity in patients with parent artery steno‐occlusion. Outcome = 0, without neurological deterioration; outcome = 1, developing neurological deterioration; WMH = 0, none‐mild WMH; WMH = 1, moderate–severe WMH; PWMH = 0, none‐mild PWMH; PWMH = 1, moderate‐severe PWMH; DWMH = 0, none‐mild DWMH; DWMH = 1, moderate‐severe DWMH.

A total of 288 participants (193 males and 95 females) were included in this study, and the average age was 66.4 years. Within 7 days after anterior circulation SSI onset, 75 (26.0%) patients developed ND. In the overall anterior circulation SSI patients, those with ND tended to have a higher prevalence of PAS, a lower prevalence of moderate‐severe PWMH, and moderate–severe WMH compared with those without ND (Table 1). Similarly, in anterior circulation SSI patients without PAS, the prevalence of moderate–severe WMH (*p *= .043) and moderate–severe PWMH (*p *= .019) were lower in those with ND compared with those without ND (Figure 1). However, in anterior circulation SSI patients with PAS, the prevalence of moderate–severe WMH, PWMH, and DWMH were similar in those with and without ND.

**TABLE 1:** Baseline characteristics of the study participants according to neurological deterioration.

Characteristics*	Neurological deterioration		
	No	Yes	*p* Value
Number of participants	213	75	
Age, years	67.1 ± 12.6	64.5 ± 13.7	.139
Male, *n* (%)	145 (68.1)	48 (64.0)	.454
Current cigarette smoking, *n* (%)	60 (28.2)	18 (24.0)	.485
Current alcohol drinking, *n* (%)	11 (5.2)	7 (9.3)	.200
History of ischemic stroke, *n* (%)	16 (7.5)	5 (6.7)	.809
Hypertension, *n* (%)	131 (61.5)	52 (69.3)	.226
Diabetes mellitus, *n* (%)	65 (30.5)	22 (29.3)	.848
Hyperlipidemia, *n* (%)	68 (31.9)	33 (44.0)	.060
Parent artery steno‐occlusion, *n* (%)	57 (26.8)	30 (40.0)	.032*
Homocysteine (μmol/L)	12.5 (10.3–15.1)	12.7 (10.2–16.0)	.874
Fasting plasma glucose (mmol/L)	5.3 (4.7–6.0)	5.4 (4.9–6.6)	.194
Low‐density lipoprotein cholesterol (mmol/L)	2.7 (2.2–3.3)	3.1 (2.7–3.8)	.873
Creatinine (μmol/L)	74.0 (63.0–88.0)	77.0 (63.0–85.0)	.538
Blood urea nitrogen (mmol/L)	4.7 (4.2–5.5)	4.4 (4.0–5.4)	.327
Hemoglobin A1c (%)	6.1 (5.8–6.7)	6.0 (5.7–6.7)	.507
Uric acid (μmol/L)	302.0 (250.0–365.0)	295.0 (258.0–357.0)	.947
Platelet counts (10^9^/L)	204.0 (169.0–250.0)	216.0 (186.0–251.0)	.275
International normalized ratio	1.0 (0.9–1.0)	1.0 (0.9–1.0)	.940
Periventricular Fazekas score	1.0 (1.0–2.0)	1.0 (1.0–1.0)	.052
Deep Fazekas score	1.0 (1.0–1.0)	1.0 (1.0–1.0)	.480
Total Fazekas score	2.0 (2.0–3.0)	2.0 (1.0–3.0)	.099
Moderate–severe periventricular white matter hyperintensity, *n* (%)	71 (33.5)	16 (21.3)	.049*
Moderate–severe deep white matter hyperintensity, *n* (%)	47 (22.2)	11 (14.7)	.164
Moderate–severe white matter hyperintensity, *n* (%)	87 (41.0)	21 (28.0)	.045*
Baseline NIHSS score at admission	2 (1–3)	3 (2–6)	.003**
Onset modified Rankin scale score	2.0 (2.0–2.0)	2.0 (2.0–2.0)	.846

*Note*: Continuous variables are expressed as mean ± SD or median (interquartile range), and categorical variables are expressed as numbers (percent).

*, *p *< .05; **, *p *< .01.

### 3.2 Association of WMH severity with ND

The association between WMH severity and the risk of ND within 7 days after stroke onset is shown in Table 2. After adjustment for several important risk factors, WMH severity was not associated with ND in total patients. However, the association between WMH severity and ND could be modified by PAS (*p*_interaction_ = .029; Table 3). After multivariate adjustment, the ORs (95% CIs) of moderate–severe WMH associated with ND were 1.61 (0.50–5.19) in patients with PAS and 0.37 (0.14–0.97) in patients without PAS.

**TABLE 2:** Risk of neurological deterioration according to white matter hyperintensity severity in ischemic stroke patients with single subcortical infarction.

	Cases/participants (%)	Age‐sex adjusted		Multiple‐adjusted	
		OR (95% CI)	*P*	OR (95% CI)	*p*
White matter hyperintensity					
None–mild	54/179 (30.2)	1.00		1.00	
Moderate–severe	21/109 (19.3)	0.61 (0.33–1.14)	.123	0.62 (0.32–1.21)	.158
Periventricular white matter hyperintensity					
None–mild	59/200 (29.5)	1.00		1.00	
Moderate–severe	16/88 (18.2)	0.59 (0.31–1.13)	.113	0.55 (0.27–1.13)	.101
Deep white matter hyperintensity					
None–mild	64/229 (28.0)	1.00		1.00	
Moderate–severe	11/59 (19.0)	0.67 (0.31–1.44)	.303	0.81 (0.35–1.86)	.617
Total Fazekas score	75/288 (26.0)	0.90 (0.70–1.15)	.401	0.88 (0.67–1.15)	.344
Periventricular Fazekas score	75/288 (26.0)	0.76 (0.50–1.14)	.187	0.69 (0.44–1.08)	.106
Deep Fazekas score	75/288 (26.0)	0.98 (0.64–1.49)	.918	0.97 (0.61–1.54)	.895

*Note*: Multiple‐adjusted: Adjusted for age, sex, current smoking, alcohol consumption, baseline NIHSS score at admission, mRS score at onset, hypertension, diabetes mellitus, hyperlipidemia, history of ischemic stroke, and parent artery steno‐occlusion.

*, *p* < .05; **, *p *< .01.

**TABLE 3:** Multivariate adjusted odds ratio and 95% confidence interval of neurological deterioration for white matter hyperintensity in patients with or without parent artery steno‐occlusion.

Variables	Patients without parent artery steno‐occlusion		Patients with parent artery steno‐occlusion		*p*_interaction_
	OR (95% CI)	*p*	OR (95% CI)	*p*	
**White matter hyperintensity**					.029*
None–mild	1.00		1.00		
Moderate–severe	0.37 (0.14–0.97)	.043*	1.61 (0.50‐5.19)	.428	
**Periventricular white matter hyperintensity**					.029*
None–mild	1.00		1.00		
Moderate–severe	0.26 (0.08–0.80)	.019*	1.39 (0.41–4.65)	.596	
**Deep white matter hyperintensity**					0.504
None–mild	1.00		1.00		
Moderate–severe	0.78 (0.25–2.41)	.665	0.97 (0.43–2.21)	.435	
**Total Fazekas score**	0.76 (0.53–1.09)	.138	1.13 (0.68–1.89)	.633	0.093
**Periventricular Fazekas score**	0.44 (0.23–0.83)	.011*	1.45 (0.62–3.36)	.390	0.024*
**Deep Fazekas score**	0.94 (0.51–1.72)	.840	0.56 (0.13–2.39)	.950	0.395

*Note*: Adjusted for age, sex, current smoking, alcohol consumption, baseline NIHSS score at admission, mRS score at onset, hypertension, diabetes mellitus, hyperlipidemia, and history of ischemic stroke.

Abbreviations: OR, odds ratio; CI, confidence interval.

*, *p* <.05; **, *p* <.01.

### 3.3 Associations of PWMH and DWMH severity with ND

We further investigated the association of PWMH and DWMH severity with ND and found that PAS mainly modified the association of ND with PWMH severity (*p*_interaction_ = .029; Table 3) but not DWMH severity. After adjustment for potential confounders, the ORs of moderate–severe PWMH were 0.26 (95% CI, 0.08–0.80) for ND, and the ORs of moderate–severe DWMH were 0.78 (95% CI, 0.25–2.41) for ND in patients without PAS (Table 3). Furthermore, there was an interactive effect between PAS and periventricular Fazekas score on the risk of ND (*p*_interaction_ = .024), with OR of periventricular Fazekas score being 0.44 (95% CI, 0.23–0.83) for ND among patients without PAS.

### 3.4 Incremental prognostic value of WMH severity in patients with anterior circulation SSI

We examined whether adding WMH severity to the conventional risk factors improved the risk prediction of ND in 7 days after anterior circulation SSI. As shown in Table 4, adding WMH severity (net reclassification improvement: 48.2%, *p *= .005; integrated discrimination index: 2.5%, *p *= .004) or PWMH severity (net reclassification improvement: 36.3%, *p *= .032; integrated discrimination index: 3.0%, *p *= .002) to a model containing conventional risk factors significantly improved predictive power for ND in anterior circulation SSI patients without PAS.

**TABLE 4:** Reclassification and discrimination statistics for neurological deterioration by white matter hyperintensity severity among single subcortical infarction patients without parent artery steno‐occlusion.

Model	NRI (%)		IDI (%)	
	Estimate	*p*	Estimate	*p*
Conventional model	1.00		1.00	
Conventional model + WMH severity	48.2 (20.0–76.4)	.005**	2.5 (0.8–4.2)	.004**
Conventional model + PWMH severity	36.3 (8.3–64.3)	.032*	3.0 (1.1–5.0)	.002**
Conventional model + DWMH severity	−6.14 (−37.3–25.0)	.718	0.1 (−0.2–0.3)	.596

*Note*: The conventional model includes age, sex, current smoking, alcohol consumption, baseline NIHSS score at admission, mRS score at onset, hypertension, diabetes mellitus, hyperlipidemia, and history of ischemic stroke.

Abbreviations: NRI, net reclassification index; IDI, integrated discrimination improvement.

*, *p *< .05; **, *p *< .01.

## 4 DISCUSSION

To our knowledge, this is the first study to investigate the association between WMH severity and ND in patients with anterior circulation SSI according to the presence or absence of PAS. In the present study, we found that the association between WMH severity and ND among patients with anterior circulation SSI was appreciably modified by PAS. Namely, WMH was associated with ND within 7 days only in anterior circulation SSI patients without PAS. Furthermore, we noted that PAS mainly modified the association between PWMH severity and ND among patients with anterior circulation SSI.

In our study, we found no association between WMH severity and ND in total patients with anterior circulation SSI. Interestingly, there was a significant interaction between WMH severity and PAS, and WMH severity might be an independent protective indicator for ND in patients without PAS, which was in line with our hypothesis based on the previous studies in SSI patients. (Cannistraro et al., 2019; Caplan, 1989; Hilal et al., 2017; Nam et al., 2021; Yamamoto et al., 2011; Zhang et al., 2014)

In addition, we found that PAS mainly modified the association between PWMH severity and ND among patients with anterior circulation SSI, and there was an inverse association of PWMH grades with the risk of ND in patients without PAS. Moreover, adding WMH severity or PWMH severity to established risk factors could improve the risk reclassification of neurological outcomes in anterior circulation SSI patients without PAS. Given the high ND rates after SSI and the limited predictive power of established prognostic factors (Whiteley et al., 2009), it is urgent for us to accurately identify prognostic factors providing additional information for clinicians to help make decisions regarding stroke management and therapeutic strategies. Our findings had important clinical implications for early screening of vulnerable groups for early targeted intervention.

WMH is associated with blood–brain barrier abnormalities and local inflammation (Moroni et al., 2018) and has been established as a marker for small cerebral vessel disease (Cannistraro et al., 2019; Hilal et al., 2017; Wardlaw et al., 2013). In 2017, Lee et al. investigated the association between imaging parameters and ND in consecutive 169 SSI patients and found no statistical differences between WMH and ND (Lee et al., 2017). However, an analysis of the Chinese Intracranial Atherosclerosis Study revealed a negative association between WMH and proximal SSI (Zhang et al., 2014), while proximal SSI is a predictive factor for ND in SSI patients(13). Nevertheless, severe WMH was shown to be associated with early neurological deterioration in 82 patients with SSI in the pontine (Nam et al., 2016). Therefore, the effects of WMH severity on neurological recovery after SSI remain unclear. Of note, these previous studies did not consider parent anterior circulation artery steno‐occlusion as an important confounder, which had been found to be significantly associated with WMH (Lee et al., 2011; Park et al., 2015; Zhai et al., 2018).

The reasons behind the observed association of WMH severity with ND in anterior circulation SSI patients without PAS can be understood at two levels. From the statistical perspective, this phenomenon arose due to the neglected interaction effect between PAS and WMH with the risk of ND in anterior circulation SSI patients. Previous reports suggested that anterior circulation PAS can predict the risk of ND (Etherton et al., 2019; Nam et al., 2021; Park et al., 2015), as well as play a vital role in the modification of the WMH severity (Lee et al., 2011; Park et al., 2015; Zhai et al., 2018). As for why WMH severity can show its predictive ability for ND in anterior circulation SSI patients without PAS, it can be simply understood that WMH severity can show its predictive ability for ND without the influence of PAS in SSI patients without PAS. From the potential pathophysiological mechanisms perspective, anterior circulation PAS can cause a certain degree of WMH, for both PAS itself (the one with the ability to lead perforators hypoperfusion) and the concomitant vascular damage factors (such as hypertension, diabetes, and so on) (Lee et al., 2011; Park et al., 2015; Zhai et al., 2018). At the same time, WMH is a neuroimaging marker of small vessel disease (Cannistraro et al., 2019; Hilal et al., 2017; Wardlaw et al., 2013). In addition, many studies have shown that PAS can easily lead to deterioration of neurological function compared with small vessel disease (Bang et al., 2002; Jeong et al., 2015; Kang et al., 2007; Petrone et al., 2016). Therefore, we consider that the above mechanism may explain why PAS affects the negative association between WMH and ND.

This study has several important strengths. First, this is the first study to investigate the association between WMH severity and ND in SSI patients based on the presence or absence of PAS. Second, all data were collected with rigorous quality control in this study, and comprehensive information about potential confounders was collected and controlled in the multivariate models. However, several limitations of the study need to be considered. First, the patients included in this study were from a single center, so it is necessary to confirm our findings in future multicenter studies with larger sample sizes. Second, this is an observational study, and although several important potential confounders had been controlled in multivariable‐adjusted models, the possibility of residual confounding cannot be eliminated. In addition, we only assessed the WMH severity with the method of the Fazekas scale, which may cause some WMH information missed compared to the quantitative method of WMH volume.

## 5 CONCLUSION

There was a modified effect of PAS on the association between WMH severity and ND after anterior circulation SSI, which deserves more research attention. WMH was negatively associated with ND in the anterior circulation SSI patients without PAS. Further studies are warranted to replicate our findings and to clarify the potential biological mechanisms.

## AUTHOR CONTRIBUTIONS

**Yunhe Luo**: Conceptualization; methodology; formal analysis; data curation; writing—original draft; writing—review and editing. **Daosheng Wang**: Conceptualization; methodology; data curation; formal analysis; writing—original draft; writing—review and editing. **Yiming Jia**: Conceptualization; data curation; formal analysis. **Xin Gu**: Conceptualization; data curation. **Yuhan Zang**: Conceptualization; formal analysis. **Zhengbao Zhu**: Conceptualization; formal analysis. **Jin Zheng**: Conceptualization; writing—review and editing. **Ligang Huang**: Conceptualization; writing—review and editing. **Jing Zhao**: Conceptualization; investigation; funding acquisition; project administration; supervision; writing—review and editing.

## CONFLICT OF INTEREST STATEMENT

The authors declare no competing interest.

### PEER REVIEW

The peer review history for this article is available at https://publons.com/publon/10.1002/brb3.3523

## Data Availability

The data that support the findings of this study are available from the corresponding author upon reasonable request.
